# Three‐Year Changes in Cognitive and Brain Functions Among Community‐Dwelling Older Adults Who Continue Working

**DOI:** 10.1111/psyg.70175

**Published:** 2026-05-08

**Authors:** Naotoshi Kimura, Daisuke Hirano, Yoshinobu Goto, Takamichi Taniguchi

**Affiliations:** ^1^ Department of Occupational Therapy School of Health Sciences at Narita, International University of Health and Welfare Chiba Japan; ^2^ Department of Occupational Therapy School of Health Sciences, International University of Health and Welfare Tochigi Japan; ^3^ Graduate School of Health and Welfare Sciences, International University of Health and Welfare Tokyo Japan; ^4^ Department of Physiology Faculty of Medicine, School of Medicine, International University of Health and Welfare Chiba Japan

**Keywords:** brain function, ERP, older adults, prevention, work

## Abstract

**Background:**

Prevention of cognitive decline in older adults is a critical public health issue in rapidly ageing societies. Studies indicate that engagement in instrumental activities of daily living (IADL) and social participation is associated with better cognitive function. Employment incorporates elements of IADL and social participation; however, longitudinal evidence describing cognitive and brain function changes in older adults who continue working is scarce. Hence, this study aimed to longitudinally examine changes in cognitive and brain functions over a three‐year period among older adults who continued working.

**Methods:**

The participants were 23 community‐dwelling older adults aged 65 years and older who completed baseline and follow‐up assessments. Cognitive function was assessed using the Japanese version of the Montreal Cognitive Assessment (MoCA‐J), Trail Making Test Parts A and B (TMT‐A and TMT‐B) and behavioural performance on a Go/No‐go task. Brain function was evaluated using event‐related potentials (ERPs), focusing on the No‐go P3 component as an index of inhibitory control. The baseline and follow‐up data were compared using paired statistical analyses.

**Results:**

The MoCA‐J scores significantly improved at follow‐up compared with those of baseline (*p* < 0.001). In the Go/No‐go task, No‐go accuracy significantly increased (*p* = 0.017). ERP analyses revealed that the No‐go P3 amplitude significantly increased at follow‐up at the Fz (*p* < 0.001), Cz (*p* = 0.002) and Pz (*p* = 0.004) electrodes, whereas the No‐go P3 latency showed no significant change.

**Conclusions:**

Older adults who continued working displayed improvements in global cognitive function, behavioural measures of inhibitory control and associated neural activity over a three‐year period. Although causal relationships cannot be established in the absence of a non‐working comparison group, our study provides longitudinal descriptive evidence of cognitive and neural changes in older adults who remain employed.

## 1 Introduction

With the rapid growth of the older adult population worldwide, preventing age‐related cognitive decline and dementia has become a major public health challenge. Maintaining cognitive function is essential not only for supporting independent living among older adults, but also for reducing the burden on long‐term care and healthcare systems [1]. Therefore, identifying modifiable lifestyle factors that support cognitive health later in life is an urgent research priority.

Previous studies have shown that engagement in instrumental activities of daily living (IADL) and social participation is associated with better cognitive function in older adults [2, 3, 4]. Employment is a distinctive activity that integrates IADL and social participation, as it entails sustained cognitive, physical and social demands. Regarding the relationship between employment and cognitive function, continued engagement in paid work later in life has been positively associated with long‐term cognitive outcomes [5]. Furthermore, greater occupational complexity in paid employment has been suggested to enhance cognitive reserve by influencing memory, language and executive functions [6]. In line with these findings, active social engagement in older adulthood has also been linked to the preservation of executive function [7].

However, studies examining cognitive function in relation to employment status in older adults have largely focused on cognitive domains, and changes in brain function remain unclear. For example, No‐go P3 is a positive‐going waveform that emerges approximately 300–600 ms after stimulus onset and is widely used as an index of neural resource allocation during response inhibition [8, 9, 10]. Inhibitory control, a core component of executive function, plays a fundamental role in supporting higher‐order cognitive processes [11]. Moreover, inhibitory control declines relatively early in the ageing process, making it a sensitive indicator for evaluating cognitive ageing [12].

This study longitudinally examined changes in cognitive and brain functions over a three‐year period among older adults who continued working. Herein, brain function was assessed using the No‐go P3 event‐related potential (ERPs) component. By integrating behavioural measures with neurophysiological indices, we aimed to clarify how continued employment influences cognitive function and inhibitory control later in life. We hypothesised that cognitive function and inhibitory control‐related brain activity would be maintained or improve over time in this cohort of older adults who continued working.

## 2 Methods

### 2.1 Ethical Considerations

This study was approved by the Ethics Committee of the International University of Health and Welfare (Approval No: 21‐Ig‐19‐3) and all participants provided written informed consent prior to participation.

### 2.2 Participants

At baseline, 46 adults aged 65 years and older who were registered with the Otawara Silver Human Resources Center (Tochigi, Japan) were enrolled in the study. The Silver Human Resources Center is an organisation whose members are older adults who do not seek regular employment but wish to work by using their skills and experience, contributing to society, earning supplementary income and enhancing their sense of purpose and social participation. The center provides temporary and short‐term jobs closely related to daily life, which are commissioned by private companies, individual households and public institutions.

For the three‐year follow‐up assessment, participants who continued working at the center were recruited. Of the initial 46 participants, 23 individuals (21 men and two women; mean age = 74.74 ± 3.07 years) completed the follow‐up assessment and were included in the final analyses. Employment status was defined as working approximately 10 days per month or no more than 20 h per week, engaging in reception, cleaning and public facility management duties. The remaining 23 participants were not included in the follow‐up assessment because they either declined to continue participation in the research or had discontinued employment at the Silver Human Resources Center.

Individuals diagnosed with dementia, cerebrovascular disease, depression, schizophrenia, or other mental disorders at the time of recruitment were excluded; it was confirmed that they did not develop these conditions during follow‐up.

### 2.3 Measurement

#### 2.3.1 Demographic Characteristics

Basic participant characteristics included age, sex and years of education. IADL were assessed using the Japan Science and Technology Agency Index of Competence (JST‐IC). The JST‐IC evaluates the abilities required for independent and active community living among older adults, including the use of new devices, information gathering, life management and social participation [13].

#### 2.3.2 Cognitive Assessment

Cognitive function was assessed using the Japanese versions of the Montreal Cognitive Assessment (MoCA‐J) and Trail Making Test Parts A and B (TMT‐A and TMT‐B). The MoCA‐J measures global cognitive function and includes subdomains that assess visuospatial/executive function, naming, memory, attention, repetition, verbal fluency, abstraction, delayed recall and orientation [14, 15]. TMT‐A and TMT‐B assess visual attention, visual search and visual–motor coordination [16]. The same versions of the MoCA‐J and TMT‐A and TMT‐B were used for baseline and follow‐up assessments.

#### 2.3.3 Go/No‐Go Task

This study evaluated brain function using the go/no‐go task, which is a frontal‐lobe inhibitory control task. The task consisted of Go trials requiring a button press and No‐go trials requiring response inhibition. In total, 300 and 100 Go and No‐go trials were conducted, respectively. Visual stimuli (“O” and “S”) were presented in a randomised order. For half of the participants, “O” was assigned as the Go stimulus and “S” as the No‐go stimulus, whereas the assignment was reversed for the other half. Each stimulus was presented for 500 ms, followed by a 500‐ms blank black screen. The task design is based on previous studies [17]. To minimise potential order effects, the presentation order of the Go and No‐go stimuli in the Go/No‐go task was changed between baseline and follow‐up assessments. The stimuli were presented using a Multi Trigger System (Medical Try System Co., Japan). The participants were seated 60 cm from the display and instructed to respond as quickly and accurately as possible following the stimulus presentation. Behavioural outcomes included Go and No‐go accuracies, mean reaction time for Go trials and intra‐individual variability in reaction time (IIV‐RT). The IIV‐RT, an index of cognitive performance, was calculated as (standard deviation/mean reaction time) × 100 [18].

#### 2.3.4 ERP Recording and Analysis

ERPs were recorded using active electrodes placed at Fz, Cz and Pz according to the international 10–20 system, with linked earlobes as the reference electrodes. Electrooculographic activity was recorded using an electrode placed above the left eye to monitor ocular artefacts. Electroencephalographic signals were sampled at 500 Hz. Active electrodes were used with the Polymate Mini AP108 system (Miyuki Giken, Tokyo, Japan). Active electrodes integrate amplification at the electrode site, which reduces output impedance and helps minimise noise contamination from the lead wires. Because active electrode systems are designed to tolerate higher electrode–skin impedance levels, impedance was maintained below 50 kΩ during recording.

ERP data were segmented into epochs from −200 to 1000 ms relative to stimulus onset, and the No‐go P3 component was analysed. The No‐go P3 reflects inhibitory control [8, 9, 10] and is a potential indicator of subtle cognitive impairment [19]. In the present study, we focused primarily on inhibitory control and therefore on the No‐go P3 component to track potential changes in cognitive function over time. The time window for the No‐go P3 was defined as 300 to 600 ms post‐stimulus [20]. The bandpass filter was used with a high‐pass filter at 0.5 Hz and a low‐pass filter at 30 Hz. Baseline correction was applied using a 200 ms pre‐stimulus interval. Trials exceeding ±100 μV were excluded as artefacts [21]. On average, 97.69 (±6.89) artefact‐free No‐go trials per participant at baseline and 97.04 (±4.78) at follow‐up were retained.

### 2.4 Statistical Analysis

Prior to analysis, data normality was assessed using the Shapiro–Wilk test, with *p* < 0.05 serving as the criterion for determining non‐normality. Between‐group differences (characteristics of the 23 participants who completed follow‐up vs. the 23 individuals who dropped out of the study and baseline vs. follow‐up data) were assessed using a paired *t*‐test when data followed a normal distribution and the Mann–Whitney *U* test when data did not follow a normal distribution. Sex was analysed using the chi‐square test. Statistical significance was set at *p* < 0.05. Effect sizes are reported alongside the *p*‐values. For non‐parametric tests, effect sizes were calculated using *r* and interpreted as large (0.50), medium (0.30) and small (0.10) [22]. For parametric tests, effect sizes were calculated using Cohen's *d*, with thresholds defined as large (0.80), medium (0.50) and small (0.20) [22]. For the chi‐square test, effect sizes were calculated using Cramer's V and interpreted as large (0.50), medium (0.30) and small (0.10) [22]. To control for type I error arising from multiple comparisons, false discovery rate (FDR) correction was applied using the Benjamini–Hochberg procedure across all outcome measures, including cognitive (MoCA‐J, TMT‐A and TMT‐B), behavioural (Go accuracy, No‐go accuracy, reaction time and intra‐individual variability of reaction time [IIV‐RT]) and ERP measures (No‐go P3 amplitude and latency at Fz, Cz and Pz). In total, 13 statistical tests were included in the FDR adjustment. Statistical significance after correction was defined as *q* < 0.05. All analyses were performed using SPSS Statistics, version 29 (IBM Corp., Armonk, NY, USA).

## 3 Results

### 3.1 Participant Characteristics

Table 1 shows the baseline characteristics of the study continuation and dropout groups. No significant between‐group differences were found in the cognitive function test or ERP results. Table 2 shows the results for the 23 participants who completed the follow‐up survey. The JST‐IC, an indicator of daily functional competence, showed no significant change between baseline and follow‐up, indicating that the participants maintained their functional independence over the three‐year period.

**TABLE 1:** Baseline characteristics of individuals who completed follow‐up and those who dropped out.

	Continuing participants	Research dropouts	*p*	ES	*W*
Age (years)	74.74 ± 3.07	72.74 ± 3.46	0.025	*r* = 0.33	0.031
Sex (male/female)	21/2	14/9	0.035	*V* = 0.35	—
Education (years)	13.00 ± 1.97	12.09 ± 1.37	0.111	*r* = 0.24	< 0.001
JST‐IC (score)	14.04 ± 2.32	13.43 ± 3.23	0.531	*r* = 0.09	< 0.001
MoCA‐J (score)	25.83 ± 1.43	25.35 ± 2.87	0.760	*r* = 0.05	0.002
TMT‐A (s)	46.21 ± 9.48	46.96 ± 20.13	0.606	*r* = 0.08	0.025
TMT‐B (s)	93.39 ± 35.45	111.35 ± 59.61	0.423	*r* = 0.12	< 0.001
Go accuracy (300 trials)	297.00 ± 5.60	295.17 ± 9.69	0.588	*r* = 0.08	< 0.001
No‐go accuracy (100 trials)	81.04 ± 13.76	80.95 ± 14.77	0.965	*r* = 0.01	< 0.001
Reaction time (ms)	344.88 ± 54.64	361.56 ± 66.18	0.475	*r* = 0.11	< 0.001
IIV‐RT (%)	22.91 ± 3.55	23.44 ± 5.84	0.714	*d* = 0.11	0.496
Nogo P3 amplitude Fz (μV)	9.8 ± 5.9	9.0 ± 4.0	0.095	*r* = 0.26	0.015
Nogo P3 amplitude Cz (μV)	9.0 ± 4.4	9.9 ± 5.1	0.508	*d* = 0.19	0.818
Nogo P3 amplitude Pz (μV)	6.4 ± 3.1	6.9 ± 4.6	0.676	*d* = 0.13	0.826
Nogo P3 latency Fz (ms)	438.5 ± 47.5	406.4 ± 46.2	0.684	*r* = 0.06	0.043
Nogo P3 latency Cz (ms)	436.6 ± 44.8	415.2 ± 45.9	0.117	*d* = 0.47	0.653
Nogo P3 latency Pz (ms)	448.6 ± 55.3	418.8 ± 49.3	0.060	*d* = 0.63	0.854

*Note:* Data are presented as mean ± SD.

Abbreviations: Cz, central zero; ES, effect size; Fz, frontal zero; IIV‐RT, intra‐individual variability in reaction time; JST‐IC, Japan Science and Technology Agency, Index of Competence; MoCA‐J, Japanese Version of Montreal Cognitive Assessment; Pz, parietal zero; TMT‐A, trail making test part A; TMT‐B, trail making test part B; W, Shapiro–Wilk test.

**TABLE 2:** Basic information and cognitive function of the subject.

	Baseline	Three years later	*p*	ES	*q*‐value	*W*
Age (years)	74.74 ± 3.07	—	—	—	—	
Sex (male/female)	21/2	—	—	—	—	
Education (years)	13.00 ± 1.97	—	—	—	—	
JST‐IC (score)	14.04 ± 2.32	13.43 ± 3.23	0.133	*r* = 0.31	—	< 0.001
MoCA‐J (score)	25.83 ± 1.43	27.22 ± 1.90	< 0.001	*r* = 0.69	0.013	0.176
TMT‐A (s)	46.21 ± 9.48	42.96 ± 10.38	0.082	*d* = 0.38	0.152	0.963
TMT‐B (s)	93.39 ± 35.45	91.02 ± 42.07	0.951	*r* = 0.01	0.951	< 0.001
Go accuracy (300 trials)	297.00 ± 5.60	297.04 ± 5.62	0.404	*r* = 0.17	0.583	< 0.001
No‐go accuracy (100 trials)	81.04 ± 13.76	85.26 ± 13.56	0.017	*r* = 0.50	0.044	< 0.001
Reaction time (ms)	344.88 ± 54.64	349.08 ± 63.25	0.648	*r* = 0.10	0.765	< 0.001
IIV‐RT (%)	22.91 ± 3.55	21.02 ± 5.28	0.043	*d* = 0.44	0.093	0.413

*Note:* Data are presented as mean ± SD.

Abbreviations: ES, effect size; IIV‐RT, intra‐individual variability in reaction time; JST‐IC, Japan Science and Technology Agency, Index of Competence; MoCA‐J, Japanese Version of Montreal Cognitive as Assessment; TMT‐A, trail making test part A; TMT‐B, trail making test part B; W, Shapiro–Wilk.

### 3.2 Cognitive Functions

The results of the cognitive assessments, including the MoCA‐J, TMT‐A and TMT‐B scores, are listed in Table 2. Among these measures, MoCA‐J scores showed significant improvement at the three‐year follow‐up compared with those at the baseline.

### 3.3 Go/No‐Go Task Performance

The behavioural results obtained from the Go/No‐go task, including Go and No‐go accuracies, reaction time and IIV‐RT, are summarised in Table 2. After controlling for multiple comparisons using the FDR correction, No‐go accuracy remained significantly improved at follow‐up. In contrast, although IIV‐RT showed a decrease over the three‐year period, this change did not remain statistically significant after FDR correction.

### 3.4 ERP Results

**FIGURE 1:**
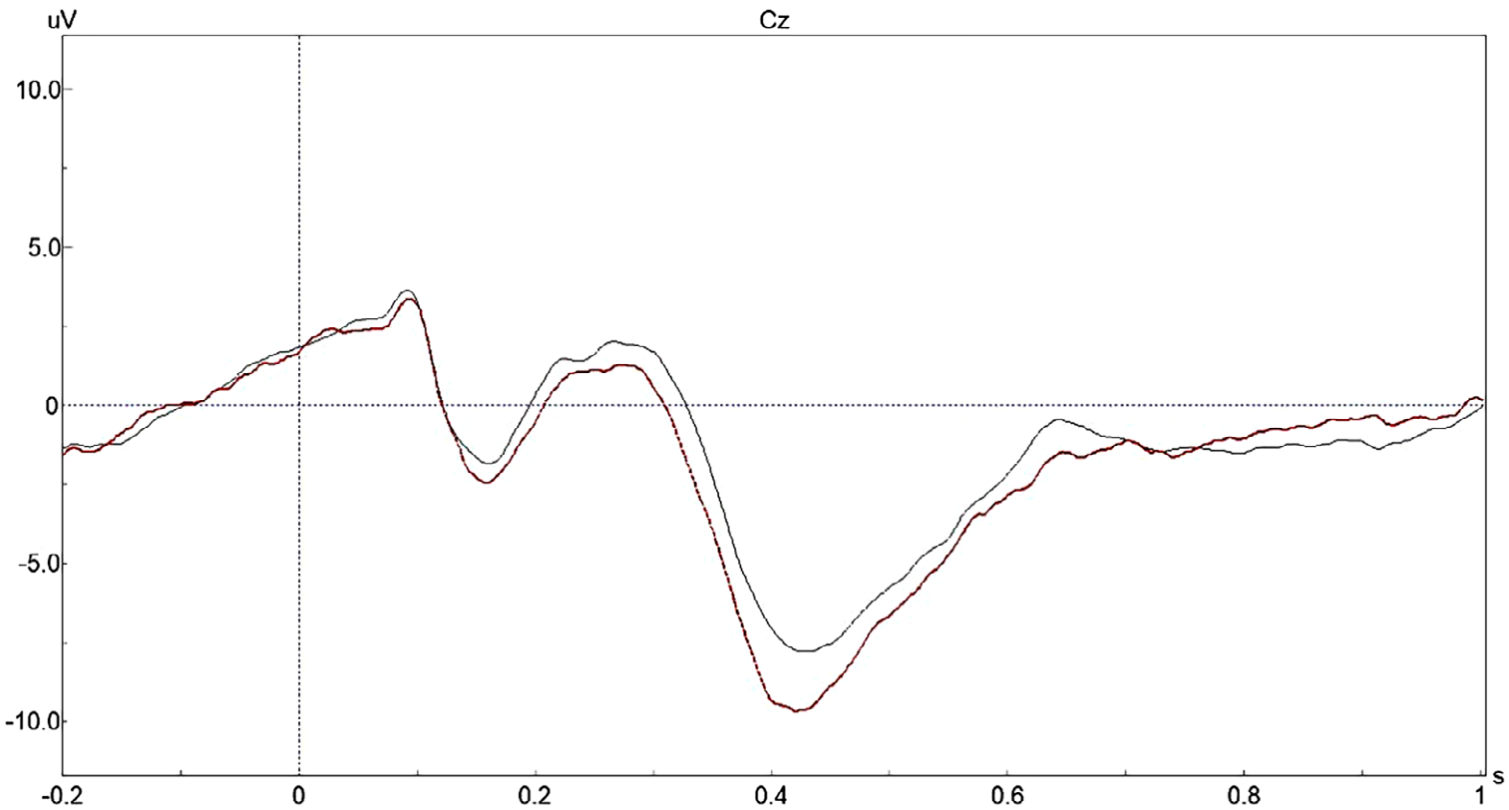
Event‐related potential waveforms at Cz during the no‐go task. *Note:* Black waveforms indicate baseline, while red waveforms indicate follow‐up.

The No‐go P3 amplitude and latency at baseline and follow‐up are presented in Table 3 and Figure 1. The No‐go P3 amplitude significantly increased at follow‐up compared to that of baseline at the Fz, Cz and Pz electrodes. In contrast, the No‐go P3 latency showed no significant differences between baseline and follow‐up.

**TABLE 3:** Comparison of Nogo P3 at baseline and after 3 years.

	Baseline	Three years later	*p*	ES	*q*‐value	*W*
Nogo P3 amplitude (μV)						
Fz	9.8 ± 5.9	11.2 ± 7.1	< 0.001	*r* = 0.88	0.006	< 0.001
Cz	9.0 ± 4.4	11.0 ± 5.3	0.002	*d* = 0.72	0.008	0.742
Pz	6.4 ± 3.1	8.2 ± 3.9	0.004	*d* = 0.67	0.013	0.174
Nogo P3 latency (ms)						
Fz	438.5 ± 47.5	430.5 ± 40.2	0.513	*r* = 0.14	0.625	< 0.001
Cz	436.6 ± 44.8	429.6 ± 35.7	0.513	*r* = 0.13	0.633	0.010
Pz	448.6 ± 55.3	444.6 ± 52.0	0.716	*d* = 0.07	0.775	0.355

*Note:* Data are presented as mean ± SD.

Abbreviations: Cz, central zero; ES, effect size; Fz, frontal zero; Pz, parietal zero; W, Shapiro–Wilk.

## 4 Discussion

In this longitudinal study, we followed community‐dwelling older adults who continued working and examined changes in cognitive and brain functions over a three‐year period. The results demonstrated improvements in global cognitive function, behavioural indices of inhibitory control and neural activity related to response inhibition. By integrating behavioural and electrophysiological measures, this study provides novel longitudinal evidence describing cognitive and neural changes in older adults who remained employed.

### 4.1 Continued Employment and Global Cognitive Function

The significant improvement in the MoCA‐J scores suggests that global cognitive function was maintained or improved over time in this cohort of older adults who remained employed. Although cognitive function generally declines with age, accumulating evidence indicates that cognitively stimulating activities, such as cognitive training, social participation and physical activity, are associated with reduced age‐related cognitive decline [23, 24, 25]. Studies indicate that employed older adults tend to exhibit higher cognitive function than those unemployed [5]. However, most studies have focused on limited cognitive domains, including memory, language and executive function [6].

The longitudinal findings of this study add to previous research by describing global cognitive function improvements over time in older adults who continued to work. However, the possibility of practise effects should be considered when interpreting these results. The MoCA score has been reported to increase upon repeated administration [26]. Because the same version of the MoCA‐J was used for both baseline and follow‐up assessments, the observed improvement may be partly attributable to familiarity with the test format and content and therefore should be interpreted with caution. Nevertheless, even considering this methodological limitation, the observed pattern of maintained or improved global cognitive performance over the three‐year period warrants further consideration. The findings may be discussed within the framework of cognitive reserve, which refers to the brain's capacity to cope with age‐related neural changes through lifelong engagement in intellectual, social and physical activities [27, 28, 29]. Employment typically involves complex cognitive processes such as planning, problem solving and decision‐making, as well as regular social interactions. Therefore, continued engagement in such cognitively and socially stimulating activities may be associated with the observed maintenance or improvement of cognitive performance in this cohort, although causal relationships cannot be established in the absence of a comparison group.

### 4.2 Inhibitory Control and Behavioural Performance

Behavioural data from the Go/No‐go task indicated a significant improvement in No‐go accuracy from baseline to follow‐up, and this effect remained statistically significant after FDR correction. Inhibitory control is a core component of executive function and is vulnerable to age‐related decline [30, 31, 32]. Improved No‐go accuracy is generally interpreted as reflecting enhanced ability to suppress inappropriate responses, which is essential for daily adaptive behaviours [33, 34, 35]. Engagement in cognitively and socially active lifestyles, including continued employment, may be associated with the maintenance of inhibitory control in later life. However, this finding should also be interpreted with caution. Whereas the presentation order of Go and No‐go stimuli was counterbalanced between baseline and follow‐up to minimise potential order effects in the present study, the potential influence of task familiarity cannot be entirely excluded.

IIV‐RT showed a decrease from baseline to follow‐up, but this change did not maintain statistical significance after FDR correction. Increased IIV‐RT is associated with cognitive impairment, including mild cognitive impairment and dementia [18], and is considered a fine‐grained cognitive marker of overall cognitive and executive function [36, 37, 38, 39]. A higher IIV‐RT is also associated with inefficient decision‐making and post‐error monitoring deficits [40, 41]. As the changes observed in this study did not survive multiple comparison corrections, the potential significance of the lower IIV‐RT should be interpreted cautiously. Additional studies with larger samples are needed to clarify whether temporal changes in IIV‐RT occur in this population.

### 4.3 Inhibitory Control Brain Function

ERP analyses revealed a significant increase in No‐go P3 amplitude, whereas no significant change in latency was observed. The P3 amplitude is a widely used index of attentional resource allocation [42]. Accordingly, the increased No‐go P3 amplitude observed in this study may reflect greater allocation of attentional resources during inhibitory control. In contrast, the No‐go P3 latency did not change significantly over time. P3 latency is thought to reflect information processing speed and has been reported to be prolonged in association with frailty, ageing and cognitive impairment [43, 44, 45]. The absence of latency prolongation suggests that inhibitory control‐related processing speed was relatively preserved over the three‐year period in this cohort. Taken together, the pattern of increased No‐go P3 amplitude and stable latency may indicate maintained or enhanced engagement of neural processes underlying inhibitory control over time. Further studies that include appropriate comparison groups are needed to clarify the extent to which these neural changes are related to continued employment.

## 5 Limitations

This study had specific limitations. First, detailed information regarding job type, work intensity and cognitive demands was not available, limiting conclusions about which aspects of employment were most beneficial. Second, the absence of a non‐working comparison group limits the ability to draw causal conclusions regarding the relationship between continued employment and cognitive or neural outcomes. In addition, participants who completed the follow‐up and continued working may represent individuals who were able to maintain a more active lifestyle. Therefore, the present findings may not be generalizable to individuals with lower cognitive function or those at risk of cognitive frailty. Third, only 23 participants completed the follow‐up, resulting in a relatively small sample size. Importantly, the sample was markedly imbalanced in terms of sex distribution (21 men and 2 women). Sex differences have been reported in cognitive ageing trajectories [46], executive function performance, and electrophysiological indices such as ERP components [47]. Additionally, employment patterns and occupational experiences differ between older men and women in Japan, which may further influence cognitive and neural outcomes [48, 49]. Therefore, the present findings primarily reflect longitudinal changes in older men who continued working and may not be generalizable to older women. Fourth, repeated administration of the same cognitive and behavioural tasks raises the possibility of practise effects. Because the same version of the MoCA‐J and the same Go/No‐go task paradigm were used at baseline and follow‐up, improvements in performance may partly reflect increased familiarity with the test procedures rather than true cognitive or neural changes. Despite our efforts to minimise order effects in the Go/No‐go task, practise‐related influences cannot be fully excluded. Fifth, although participants with a history of psychiatric disorders were excluded at recruitment, current mental state (e.g., mood, stress, or subclinical depressive symptoms) was not assessed. Therefore, the potential impact of these factors on cognitive function and neurological indicators could not be completely ruled out. Future studies should include larger samples, detailed assessments of occupational characteristics, appropriate control groups, alternate test forms when available, assessment of mental state and sex‐specific analyses to further elucidate the mechanisms that link continued employment and cognitive ageing.

## 6 Conclusion

This longitudinal study demonstrated that older adults who continued working exhibited improvements in global cognitive function, behavioural performance related to inhibitory control, and neural activity associated with response inhibition over a three‐year period. Notably, increased No‐go P3 amplitude accompanied by preserved latency suggests enhanced recruitment of neural resources for inhibitory control without a concomitant decline in information processing speed. Overall, our study provides longitudinal evidence of changes in cognitive performance and inhibitory control‐related neural activity over time in older adults who remain employed. Further research utilising data from appropriate comparison groups is needed to clarify the influence of continued employment on cognitive and brain ageing.

## Conflicts of Interest

The authors declare no conflicts of interest.

## Data Availability

Research data are not shared.
